# Long-term results of radical prostatectomy with immediate adjuvant androgen deprivation therapy for pT3N0 prostate cancer

**DOI:** 10.1186/1471-2490-14-13

**Published:** 2014-01-29

**Authors:** Yuzuri Tsurumaki Sato, Hiroshi Fukuhara, Motofumi Suzuki, Tetsuya Fujimura, Tohru Nakagawa, Hiroaki Nishimatsu, Haruki Kume, Teppei Morikawa, Masashi Fukayama, Yukio Homma

**Affiliations:** 1 Department of Urology, Graduate School of Medicine, The University of Tokyo, 7-3-1 Hongo, Bunkyo-ku, Tokyo 113-8655, Japan; 2 Department of Pathology, The University of Tokyo Hospital, 7-3-1 Hongo, Bunkyo-ku, Tokyo 113-8655, Japan

**Keywords:** Adjuvant androgen deprivation therapy, Pathological T3, Prognosis, Prognostic factor, Prostate cancer, Radical prostatectomy

## Abstract

**Background:**

Radical prostatectomy is used to treat patients with clinically localized prostate cancer, but there have been few reports of its use in locally advanced disease. We evaluated the long-term results of radical prostatectomy and immediate adjuvant androgen deprivation therapy in Japanese patients with pT3N0M0 prostate cancer.

**Methods:**

We retrospectively reviewed 128 patients with pT3N0M0 prostate cancer who underwent radical prostatectomy at our institute from 2000 to 2006. All pT3N0 patients were treated with adjuvant androgen deprivation therapy shortly after radical prostatectomy. Immediate adjuvant androgen deprivation therapy was continued for at least 5 years. Twenty-three were excluded because of preoperative hormonal therapy, missing data, or others. Death from any cause, death from prostate cancer, clinical recurrence and hormone-refractory biochemical progression were analyzed by Kaplan-Meier graphs. Relative risks of progression were estimated using Cox proportional hazards models with 95% confidence intervals.

**Results:**

The 10-year hormone-refractory biochemical progression-free survival rate was 88.3% and the cancer-specific survival rate was 96.3% after a median follow-up period of 8.2 years (range 25.6-155.6 months). Higher clinical stage (p = 0.013), higher Gleason score at biopsy (p = 0.001), seminal vesicle invasion (p = 0.003) and microlymphatic invasion (p = 0.006) were predictive factors for hormone-refractory biochemical progression by univariate analyses. Multivariate analyses identified Gleason score at biopsy (p = 0.027) and seminal vesicle invasion (p = 0.030) as independent prognostic factors for hormone-refractory biochemical progression. None of the patients with clinical T1 cancers (n = 20), negative surgical margin (n = 12), or negative perineural invasion (n = 11) experienced hormone-refractory biochemical progression.

**Conclusions:**

Radical prostatectomy with immediate adjuvant androgen deprivation therapy may be a valid treatment option for patients with pT3N0M0 prostate cancer.

## Background

Tumor cell penetration of the prostatic capsule or invasion of the seminal vesicle is recognized as locally advanced prostate cancer of pathological T3N0. Patients with pT3N0 prostate cancers have the potential to suffer from disease relapse, and radical prostatectomy alone may fail to achieve a cure. The introduction of prostate specific antigen (PSA) assays means that more patients now undergo radical prostatectomy at earlier stages. However, pT3 disease still occurs in 25–58% of clinical T1 and T2 prostate cancer patients [1-4]. Although the management of patients with pT3 prostate cancer remains controversial, some reports recommend the use of adjuvant therapies in these patients [5-9]. Few studies have reported treatment outcomes of pT3 cancers, but some clinicopathologic factors, such as higher Gleason score, higher PSA level and seminal vesicle invasion are considered to be prognostic factors associated with poorer outcome [10-13]. However, to the best of our knowledge, there have been few reports of pT3N0 patients treated with adjuvant hormonal therapy, and more outcome data and accurate information are needed for these patients. We therefore analyzed clinical data from patients with pT3N0 prostate cancer to obtain detailed information and long-term outcome data. Importantly, a pathologic diagnosis of pT3N0M0 cancer is not necessarily accurate in patients who have undergone preoperative therapy, and this study therefore only included patients who had not received any preoperative treatment.

## Methods

We retrospectively reviewed 128 patients with pT3N0M0 out of a total of 431 patients with prostate cancer who underwent radical prostatectomy at our hospital from January 2000 to December 2006. These patients were selected because immediate adjuvant hormonal therapy was applied in patients with pT3N0M0 prostate cancer during this period. Twenty-three patients were excluded from the analysis; four because of incomplete data, 12 because they had received preoperative hormonal therapy and seven because they had received bicalutamide only or estramustine as hormonal therapy. Data from the remaining 105 patients were analyzed. Bilateral obturator lymph nodes were dissected in all patients at radical prostatectomy. Immediate adjuvant androgen deprivation therapy was started within 12 weeks of radical prostatectomy. Undetectable PSA levels or PSA nadir were not required to be confirmed following radical prostatectomy. Clinical diagnosis was defined according to the 2009 TNM guidelines based on a digital rectal examination (DRE), transrectal ultrasonography, biopsy results, computed tomography scans and/or magnetic resonance imaging, and bone scintigraphy. All specimens were reviewed by a single pathologist. After radical prostatectomy, patients were followed-up at 1-month intervals for the first 3 months following surgery, then at 3-month intervals for 5 years, and finally at 3–6-month intervals thereafter. Follow-up examinations included measurement of PSA levels, and a DRE, computed tomography scan, magnetic resonance imaging or bone scintigraphy in the event of suspected disease recurrence. Immediate adjuvant therapy included surgical orchiectomy, administration of luteinizing hormone-releasing hormone (LHRH) analogs, and combined androgen blockade consisting of orchiectomy or an LHRH analog together with anti-androgens. Immediate adjuvant androgen deprivation therapy was continued for at least 5 years after radical prostatectomy. Salvage additive or altered hormonal therapies were initiated when the PSA level rose rapidly by >0.4 ng/ml, or when it rose consistently by >0.2 ng/ml for more than three consecutive visits. Salvage additive or altered hormonal therapy included: 1) combined androgen blockade consisting of orchiectomy or LHRH analog with bicalutamide; 2) suspension of bicalutamide to confirm the effects of anti-androgen withdrawal; 3) estramustine; and 4) dexamethasone.

Hormone-refractory biochemical progression was defined as a PSA level >0.2 ng/ml despite the above hormonal therapies. Clinical recurrence was defined as recognizable disease relapse on imaging examination. Clinical recurrence was treated by salvage radiation therapy. This study was approved by the Ethics Committee, Graduate School of Medicine and Faculty of Medicine, The University of Tokyo.

The study end points were death from any cause, death from prostate cancer, clinical recurrence, and hormone-refractory biochemical progression. These end points were analyzed by plotting Kaplan-Meier graphs and comparing them according to each clinicopathologic factor using log-rank tests. Relative risks for hormone-refractory biochemical progression according to each clinicopathologic factor were estimated using the Cox proportional hazards models with 95% confidence intervals. All statistical analyses were performed using JMP version 9 (SAS Institute, Cary, NC, USA) and differences were considered statistically significant at p < 0.05. The following clinicopathologic factors were evaluated: age at radical prostatectomy, preoperative PSA level, preoperative T stage (clinical stage), Gleason score of the biopsy specimen, seminal vesicle invasion (representing stage pT3b), surgical margin of operation specimen, microlymphatic invasion, microvascular invasion, perineural invasion, and Gleason score.

## Results

The clinical and pathological data for all 105 patients are shown in Table 1. The median age at surgery was 67.0 years and the median preoperative PSA level was 15.1 ng/ml (range 3.5–160.7, with lower and upper quartile values of 8.18 and 24.9). The median number of lymph nodes removed was 7.0 (range 2–19). A total of 43% of patients were underestimated preoperatively as having stage T1 (n = 20; 19.0%) or T2 (n = 25; 23.8%) tumors. Regarding the Gleason score at biopsy, 38 (36.2%) patients had a score of ≥8. Seminal vesicle invasion (pT3b) was detected in 42 patients (40.0%). Microlymphatic and microvascular invasions were detected in 33 (31.4%) and 51 (48.6%) patients, respectively. Immediate adjuvant androgen deprivation therapy consisted of androgen suppression with orchiectomy (n = 17), LHRH analog (n = 64), combined androgen blockade with orchiectomy or LHRH analog and bicalutamide or other anti-androgens (n = 24). Three patients changed their hormonal therapies during follow-up because of adverse events.

**Table 1:** Patient characteristics (n = 105)

**Parameter**		***n***	**(%)**
Age, years (median 67.0)	<65	37	35.2
	≥65	68	64.8
PSA, ng/ml [median 14.3 (2.4 − 160.7)]	<10	35	33.3
	10 ~ 20<	36	34.3
	20 ~ 50<	23	21.9
	≥50	11	10.5
Clinical stage	T1	20	19.0
	T2	25	23.8
	T3	59	56.2
	T4	1	1.0
Gleason score at biopsy	5 ~ 6	34	32.4
	7	33	31.4
	≥8	38	36.2
Seminal vesicle invasion	−	63	60.0
	+	42	40.0
Surgical margin	−	12	11.4
	+	93	88.6
Microlymphatic invasion	−	72	68.6
	+	33	31.4
Microvascular invasion	−	54	51.4
	+	51	48.6
Perineural invasion	−	11	10.5
	+	94	89.5
Gleason score at prostatectomy	5 ~ 6	16	15.2
	7	54	51.4
	≥8	35	33.3

The median follow-up period was 98.7 months (range 25.6–155.6). During the follow-up period, eleven patients (10.5%) experienced hormone-refractory biochemical progression. Seven patients experienced clinical recurrence and received salvage radiation therapy to clinically recurrent foci. Three (2.9%) patients died of prostate cancer and eight (7.6%) died of other causes. The 5- and 10-year cancer-specific survival rates were 98.1 and 96.3%, respectively. The 5- and 10-year hormone-refractory biochemical progression-free survival rates were 94.3 and 88.3%, with 5- and 10-year clinical recurrence-free survival rates of 96.0 and 93.0%, respectively. The 10-year estimated overall survival rate was 85.7%.

Table 2 shows the hormone-refractory biochemical progression-free survival rates calculated from Kaplan-Meier graphs, according to each clinicopathologic parameter. Univariate analyses using Cox proportional hazard models indicated that higher clinical stage (p = 0.013), higher Gleason score at biopsy (p = 0.001), seminal vesicle invasion (p = 0.003) and microlymphatic invasion (p = 0.006) were predictive factors for hormone-refractory biochemical progression (Table 2). Multivariate analyses identified Gleason score at biopsy and (p = 0.027) seminal vesicle invasion (p = 0.030) as independent prognostic factors for hormone-refractory biochemical progression (Table 2).

**Table 2:** Hormone refractory biochemical progression-free survival according to each clinicopathological parameter

**Univariate and multivariate analyses for hormone refractory biochemical-progression free survival rates**									
**Parameter**		**Hormone refractory biochemical progression-fee survival rates**		**Univariate analysis**			**Multiivariate analyses**		
		**5 years**	**10 years**	**HR†**	**95% CI††**	***p***	**HR†**	**95% CI††**	***p***
Age, years (median 67.0)	<65	94.6	89.9						
	≥65	94.1	87.6			0.687			
PSA, mg/ml [median 15.1 (3.5−160.7)]	<10	97.1	97.1	1					
	10-20<	88.9	80.8	6.18	1.1, 116.8	0.043			
	20-50<	100.0	100.0	0.0	0,.	0.315			
	≥50	90.9	60.6	12.9	1.9, 254.0	0.008			
Clinical stage	T1,2	100.0, 100.0	100.0, 88.9	1			1		
	T3~4	90.0	84.8	7.47	1.4, 137.1	0.013*	3.65	0.7, 68.6	0.161
Gleason score at biopsy	5~7	98.5	98.3	1			1		
	≥8	86.8	70.3	8.38	2.2, 55.0	0.001*	4.73	1.2, 31.7	0.027*
Seminal vesicle invasion	−	98.4	98.4	1			1		
	+	88.1	73.1	7.42	1.9, 48.7	0.003*	4.53	1.1, 30.1	0.030*
Surgical margin	−	100.0	100.0						
	+	93.6	86.7			0.183			
Microlymphatic invasion	−	97.2	95.0	1			1		
	+	87.9	74.4	5.33	1.5, 24.7	0.006*	2.18	0.9, 12.8	0.140
Microvascular invasion	−	96.3	90.5						
	+	92.2	85.6			0.468			
Perineural invasion	−	100.0	100.0						
	+	93.6	86.7			0.242			
Gleason score at prostatectomy	5−7	95.7	89.6						
	≥8	91.4	71.4			0.114			

†Hazard ratio by Cox proportional-hazard models

††Confidence interval

## Discussion

Despite widespread use of PSA measurement, pT3N0M0 prostate cancer still occurs in 25–58% of clinical T1 and T2 prostate cancer patients [1-4]. In the current study, about half of the patients (43%) were also understaged preoperatively as having organ-confined disease. Although pT3N0M0 prostate cancer is not rare, there have been few reports of treatment outcomes in these patients. The optimal postsurgical management for patients with such unfavorable pathological features remains questionable. We therefore analyzed clinical data from patients with pT3N0M0 prostate cancer to obtain detailed information and long-term outcome data.

The patients in this study achieved 5- and 10-year cancer-specific survival rates of 98.1 and 96.3%, respectively, and 5- and 10-year hormone-refractory biochemical progression-free survival rates of 94.3 and 88.3%, respectively. In a previous study, Inagaki et al. reported 1- and 3-year biochemical progression-free survival rates of 53.7 and 34.1% in 106 patients with pT3N0M0 prostate cancer treated with radical prostatectomy alone, after a mean follow-up of 1.5 years [10]. Delongchamps et al. and Briganti et al. reported 5-year biochemical progression-free survival rates of 48 and 45.0% in 147 and 500 patients with pT3N0M0 prostate cancer, respectively, treated with radical prostatectomy alone, after median follow-up periods of 5 and 3.9 years [11,12]. Thompson et al. reported 10-year metastasis-free survival rates and overall survival rates of 61 and 66% in 211 patients with pT3N0M0 prostate cancer treated with radical prostatectomy, even after salvage radiotherapy [5]. Single-modality therapy involving surgery alone might be of limited use in patients with stage pT3N0M0 prostate cancer, and a multimodal approach may be more beneficial. Three studies found that adjuvant radiotherapy after radical prostatectomy reduced the risk of subsequent biochemical recurrence in randomized clinical trials [5-7]. Thompson et al. reported a survival benefit of adjuvant radiotherapy, with a 10-year estimated survival rate of 74% in 214 patients with pT2-3 N0 prostate cancer treated with adjuvant radiotherapy, compared with 66% in 211 cases treated with surgery alone, after median follow-up period of 12.5 years [5,6]. This cohort included patients with pT3N0 and pT2N0 with positive surgical margins, and patients were allowed to receive salvage hormonal therapy during the follow-up period. Regarding the combination of radical prostatectomy and adjuvant hormonal therapy, Dorff et al. reported favorable outcomes in an interim report of a prospective randomized trial of 481 patients with pT2-3 N0-1 prostate cancer, including 61% of T3 and 16% of N1 patients [14]. Although longer observation periods are awaited, they reported 5-year biochemical progression-free and overall survival rates of 92.5 and 95.5%, respectively, in patients treated with adjuvant hormonal therapy consisting of goserelin and bicalutamide, after a median observation period of 4.4 years. Some studies reported encouraging results in more challenging patients with more severe pathological stages. Spahn et al. reported on 173 patients with pT3N0-1 tumors, including 43.3% of N1, who had undergone prostatectomy [15]. They reported an 8-year cancer-specific survival rate of 86.3% and an overall survival rate of 77.3% after a median observation period of 5.7 years in patients treated with adjuvant hormonal therapy comprising an LHRH analog with or without flutamide. Siddiqui et al. reported an advantage of adjuvant hormonal therapy with an LHRH analog, bilateral orchiectomy, or anti-androgens in a retrospective study of 191 pT3bN0M0 prostate cancer patients [9]. They found that, although the overall survival rate was similar to that in the matched control cohort, the biochemical progression-free and cancer specific survival rates were improved, with 10-year biochemical progression-free and cancer-specific survival rates of 60 and 94%, respectively, after a median follow-up of 10 years [9]. In accordance with this previous report, subgroup analyses of pT3bN0 patients in the current study demonstrated excellent outcomes, with 5- and 10-year cancer-specific survival rates of 95.1 and 90.8%, respectively. These results indicate that the combination of radical prostatectomy and adjuvant androgen deprivation therapy may produce excellent outcomes in patients with pT3N0M0 prostate cancer.

The current study achieved a 10-year cancer-specific survival rate of 96.3% and a 10-year estimated overall survival rate of 85.7% after a median follow-up period of 8.2 years. These survival rates were higher than those in previous reports, which may require an explanation. Immediate commencement of adjuvant androgen deprivation therapy after radical prostatectomy, and its comparatively long duration (at least 5 years), may have contributed to the beneficial effect. Supportive treatment strategies, such as prompt adjustment or alteration of hormonal therapy in the event of a slight increase in PSA levels, may also have improved the treatment efficacy. It is also possible that Japanese men are more sensitive than other ethnic groups to hormonal therapy after radical prostatectomy. Akaza et al. reported 5- and 10-year cancer-specific survival rates of 90 and 69%, respectively, in 68 Japanese patients with clinical T3N0M0 tumors who were treated with hormonal therapy alone [16]. However, Ueno et al. reported 5- and 8-year progression-free survival rates of 59.8 and 48.1%, respectively, in 245 Japanese patients with clinical T3N0M0 cancers treated with combined androgen blockade (63.5%) or castration [17].

The Asia Consensus Statement 2013 in the NCCN Clinical Practice Guidelines in Prostate Cancer states that androgen deprivation is a candidate treatment option for post-radical prostatectomy recurrence in Asian patients negative for distant metastasis. The results of the current study suggest that a treatment strategy consisting of radical prostatectomy and immediate adjuvant androgen deprivation therapy may offer favorable cancer control in Japanese patients with pT3N0M0 prostate cancer. This strategy was also feasible and well tolerated. Immediate adjuvant androgen deprivation therapy thus represents an attractive option for patients with pT3N0M0 prostate cancer.

Few studies have reported on prognostic factors in patients with pT3 prostate cancer. The current study found that higher clinical stage, higher Gleason score at biopsy, and seminal vesicle and microlymphatic invasion were unfavorable factors, and multivariate analyses identified seminal vesicle invasion and Gleason score at biopsy as independent prognostic factors for hormone-refractory biochemical progression. Interestingly, no patients with clinical T1 tumors (n = 20), negative surgical margin (n = 12), or negative perineural invasion (n = 11) experienced hormone-refractory biochemical progression. In partial agreement with our results, previous studies identified Gleason score, PSA, seminal vesicle invasion and lymphovascular invasion as prognostic predictors in patients with pT3N0 stage prostate cancer undergoing radical prostatectomy [10-13].

The limitations of this study included its retrospective nature and the relatively small sample size. Further investigations, including prospective studies, are needed to compare the additive effects of multimodal therapies in patients with pT3N0, to allow the better selection of patient populations most likely to benefit from treatment. The current study indicated a significant hazard ratio for seminal vesicle invasion or with higher Gleason score at biopsy, suggesting that patients with pT3b or with higher Gleason score may be the leading candidates for such studies.

These findings were based on pathologic results. The majority of the patients included in the study were considered to have lower grade and stage at diagnosis, and T3N0 was only diagnosed after radical prostatectomy. These results suggest that radical prostatectomy is a reasonable option for the initial treatment of prostate cancer, and allow for the better selection of patients who will require additional therapies.

## Conclusions

Radical prostatectomy with immediate adjuvant androgen deprivation therapy may be a valid treatment option for patients with pT3N0 prostate cancer.

## Competing interests

The authors declared that they have no competing interests.

## Authors’ contributions

YTS made substantial contributions to conception and design, analysis and interpretation of data and was involved in drafting the manuscript. HF made substantial contributions to conception and design, analysis and interpretation of data and was involved in revising it critically for important intellectual content. MS, TF, TN and HN made substantial contributions to acquisition of data. HK made substantial contributions to conception and design and helped to draft the manuscript. TM and MF evaluated the pathological specimens in a manner blinded to the clinical information. YH conceived and supervised the study, helped to draft the manuscript and was involved in revising it critically for important intellectual content. All authors read and approved the final manuscript.

## Pre-publication history

The pre-publication history for this paper can be accessed here:

http://www.biomedcentral.com/1471-2490/14/13/prepub
